# Identification of Key Genes Related to CD8+ T-Cell Infiltration as Prognostic Biomarkers for Lung Adenocarcinoma

**DOI:** 10.3389/fonc.2021.693353

**Published:** 2021-09-28

**Authors:** Minjun Du, Yicheng Liang, Zixu Liu, Xingkai Li, Mei Liang, Boxuan Zhou, Yushun Gao

**Affiliations:** Department of Thoracic Surgery, National Cancer Center/National Clinical Research Center for Cancer/Cancer Hospital, Chinese Academy of Medical Sciences and Peking Union Medical College, Beijing, China

**Keywords:** lung adenocarcinoma, immune microenvironment, CD8+ T cell, bioinformatics analysis, multiplex immunohistochemistry

## Abstract

**Background:**

CD8+ T cells are one of the central effector cells in the immune microenvironment. CD8+ T cells play a vital role in the development and progression of lung adenocarcinoma (LUAD). This study aimed to explore the key genes related to CD8+ T-cell infiltration in LUAD and to develop a novel prognosis model based on these genes.

**Methods:**

With the use of the LUAD dataset from The Cancer Genome Atlas (TCGA), the differentially expressed genes (DEGs) were analyzed, and a co-expression network was constructed by weighted gene co-expression network analysis (WGCNA). Combined with the CIBERSORT algorithm, the gene module in WGCNA, which was the most significantly correlated with CD8+ T cells, was selected for the subsequent analyses. Key genes were then identified by co-expression network analysis, protein–protein interactions network analysis, and least absolute shrinkage and selection operator (Lasso)-penalized Cox regression analysis. A risk assessment model was built based on these key genes and then validated by the dataset from the Gene Expression Omnibus (GEO) database and multiple fluorescence *in situ* hybridization experiments of a tissue microarray.

**Results:**

Five key genes (MZT2A, ALG3, ATIC, GPI, and GAPDH) related to prognosis and CD8+ T-cell infiltration were identified, and a risk assessment model was established based on them. We found that the risk score could well predict the prognosis of LUAD, and the risk score was negatively related to CD8+ T-cell infiltration and correlated with the advanced tumor stage. The results of the GEO database and tissue microarray were consistent with those of TCGA. Furthermore, the risk score was higher significantly in tumor tissues than in adjacent lung tissues and was correlated with the advanced tumor stage.

**Conclusions:**

This study may provide a novel risk assessment model for prognosis prediction and a new perspective to explore the mechanism of tumor immune microenvironment related to CD8+ T-cell infiltration in LUAD.

## Introduction

Lung adenocarcinoma (LUAD) is the most common type of lung cancer, accounting for 40% of all lung cancers (1–3). In recent years, the development of immunotherapy has changed the landscape of non-small cell lung cancer (NSCLC) therapy (4–6). Notably, the immunotherapy effects mainly rely on the immune responses, which are significantly influenced by the tumor microenvironment (7, 8). CD8+ T cells are central effector cells in the tumor microenvironment, and previous studies have reported that highly infiltrating CD8+ T cells are beneficial to prognosis in most tumors, including LUAD (9–14). However, the mechanism of CD8+ T-cell infiltration in the tumor microenvironment in LUAD is still unclear. Therefore, identifying novel biomarkers related to CD8+ T-cell infiltration may help explore the immune infiltration mechanism in LUAD.

With the rapid development of bioinformatics, new tools have arisen to identify novel biomarkers (15–21). For example, weighted gene co-expression network analysis (WGCNA) is an effective tool that mines related patterns between genes to identify relevant modules and hub genes in cancer (16), and it has been widely used to find biomarkers at the transcriptional level (17, 18). Another bioinformatics tool, namely, Cell Type Identification by Estimating Relative Subsets of RNA Transcripts (CIBERSORT), is used to quantify the cellular composition of immune cells using a deconvolution algorithm based on gene expression data (19). This algorithm has been successfully used to approximate the level of immune cell infiltration in various cancers, such as prostate cancer and renal cancer (20, 21).

Previously, many studies have focused on exploring the immune-genomic biomarkers, which may direct immunotherapy. For example, tumor mutational burden (TMB) may be a preferable choice for directing the first-line immuno-oncology agent management of advanced non-oncogene-addicted NSCLC patients (22, 23). However, fewer studies are focusing on exploring prognostic biomarkers from the aspect of immune cell infiltration, which could be used not only to estimate prognosis but also to direct immunotherapy. In this study, to identify the hub genes related to CD8+ T immune cell infiltration and potential biomarkers of LUAD, we first used WGCNA to obtain differentiated gene expression modules based on gene expression data in The Cancer Genome Atlas (TCGA) database. The CIBERSORT algorithm was used to calculate the T-cell compositions of the samples. Those important modules and hub genes related to CD8+ T-cell infiltration were identified by correlation analysis of the WGCNA and CIBERSORT algorithm results. Furthermore, the immune and clinical characteristics of the hub genes were verified, and a risk score model based on the hub genes was built, which were significantly related to the prognosis of LUAD after least absolute shrinkage and selection operator (Lasso) regression analysis and multivariable Cox analysis. The model’s performance was evaluated using receiver operating characteristic (ROC) curves, calibration curve, and stratification analysis. Gene Expression Omnibus (GEO) datasets were then conducted for external validation. Furthermore, we performed multiple fluorescence *in situ* hybridization of 98 LUAD tissues and 82 adjacent tissues to further verify the results of bioinformatics analysis. This is the first time that the WGCNA and CIBERSORT algorithm were used to identify the relevant biomarkers of infiltration of CD8+ T cells in LUAD and to further build a LUAD prognosis prediction model.

## Materials and Methods

### Data Collection

Expression and clinical data (478 cases of LUAD and 51 normal lung tissues) were downloaded from UCSC TCGA (https://gdc.xenahubs.net/download/TCGA-LUAD.htseq_counts.tsv.gz; https://gdc.xenahubs.net/download/TCGA-LUAD.GDC_phenotype.tsv.gz; https://gdc-hub.s3.us-east-1.amazonaws.com/download/TCGA-LUAD.survival.tsv). The Ensembl database (http://www.ensembl.org/info/data/ftp/index.html) was used for downloading human gtf files (Homo_sapiens.GRCh38.99.gtf.gz) and acquiring symbol data. The validation dataset (GSE72094) containing 393 cases of LUAD was downloaded from the GEO database through the R package “GEOquery.”

### Analysis of the Differential Gene Expression

Differentially expressed gene (DEG) analysis was performed using the R package “edgeR” and visualized by volcano plot and heatmap. The heatmap and the volcano plot were done with the R packages “pheatmap” and “EnhancedVolcano,” respectively. The threshold of DEGs was set at |logFC| >1 and false discovery rate <0.05.

### Co-Expression Network Construction by Weighted Gene Co-Expression Network Analysis

With the use of the R package “WGCNA,” a weight co-expression network was constructed based on the expression value of 8,807 DEGs (16). According to Pearson’s correlation value between paired genes, a similarity matrix containing the expression levels of individual transcripts was built. Then, based on the equation, adjacency between the paired genes = |Pearson’s correlation between the paired genes|^β^, the similarity matrix was converted into an adjacency matrix. The parameter β could amplify differences of correlation between genes. When β = 4, the adjacency matrix was converted into a topological overlap matrix. Finally, we used a bottom-up algorithm to classify genes with similar expression patterns into different modules.

### Construction of Module Feature Relationships

With the use of the R package “CIBERSORT,” the proportions of 22 types of immune cells in the samples were deduced according to the expressions of genes. The expression of signature genes was extracted to form a signature gene expression matrix. Combined with the known immune cell signature, the immune cell proportions of samples were calculated using “CIBERSORT,” and finally, the proportions of relevant subtypes of T cells were extracted. Furthermore, the correlations between genes of modules in WGCNA and the subtypes of T cells were calculated by Pearson’s test. The modules most significantly correlated with CD8+ T cells were selected for the subsequent analyses.

### Enrichment Analysis of Functions and Signaling Pathways

The enrichment of functions and signaling pathways of genes in the identified hub module was conducted using the R package “clusterProfiler,” and the threshold was p-value <0.05 and q-value <0.2. After the enrichment pathways were determined, a bubble map was plotted.

### Identification of Hub Genes Associated With Infiltration of CD8+ T Cells and Prognosis

To further determine the central nodes in the modules related to immune cell infiltration, we imported the co-expression network of relevant modules of WGCNA into Cytoscape (https://cytoscape.org/) and then screened the genes with high nodes according to a different degree. Furthermore, approximately one-third of the total genes in the modules were selected as hub genes according to the threshold of degree = 260. Meanwhile, all genes in the hub module were imported into the STRING database (https://string-db.org/), and then a protein–protein interaction (PPI) network was constructed. The network was imported into Cytoscape to search central nodes, and approximately one-third of the total genes were selected as hub genes when the degree = 5. Finally, a Venn plot integrated the WGCNA and STRING database results to identify the hub genes. After acquiring the hub genes, we used univariate Cox regression analyses to preliminarily screen the hub genes associated with the prognosis, and the genes with statistical significance (p < 0.05) underwent Lasso-penalized Cox regression analysis for further dimension reduction. Genes with statistical significance (p < 0.05) in Lasso-penalized Cox regression analysis were considered key hub genes associated with CD8+ T-cell infiltration and selected for the subsequent analyses.

### Construction and Validation of a Prognostic Risk Model

Based on key hub genes associated with CD8+ T-cell infiltration, a prognostic risk model was constructed. The risk score was calculated as follows: risk score = (β_A_ × gene A expression) + (β_B_ ×gene B expression) ··· + (β_N_ × gene N expression). To evaluate the model’s performance, the “survival” package was used to draw a calibration curve, and the “survivalROC” package was used to draw ROC curves. An area under the ROC curve (AUC) >0.6 was considered as a good performance of the model. Patients were divided into high-risk or low-risk groups according to the median value of the risk score, and the Kaplan–Meier method with log-rank test was used to test the prognostic significance of the risk score. p < 0.05 was considered statistically significant.

The prognostic model was then validated by the GSE72094 dataset from the GEO database. The risk scores of samples were calculated as the formula shown above. The ROC curve and the calibration curve were drawn to evaluate the performance, as well as the Kaplan–Meier method and the log-rank test were used to compare the prognostic significance between the high-risk group and the low-risk group. Because EGFR mutation status is critical in LUAD, we estimated the prognostic model in wild-type and mutation-type EGFR samples in the construction and validation cohorts.

### Correlation Between Key Hub Genes and Subtypes of Immune Cells

The correlations of key hub genes and immune cell subtypes were calculated online using the TIMER database (http://timer.cistrome.org/). Then, together with the correlation data, a heatmap was plotted to show the correlations, and scatter diagrams were shown for different key hub genes.

### External Validations of Protein Expression by Multicolor Immunofluorescence

The lung cancer tissue array with 82 pairs of matched cancerous and adjacent tissues, as well as an additional 16 cases of cancer tissues (HLugA180Su07), was obtained from Shanghai Outdo Biotech. To assess the expressions of key hub genes, multicolor immunofluorescence (mIHC) was performed using an Opal 7-color fluorescent IHC kit (PerkinElmer) combined with automated quantitative analysis (AQUA; Genoptix). First, the concentrations and order of the five antibodies were optimized, and a spectral library was built based on single-stained slides. The slides were first deparaffinized with xylene and ethanol, and antigen retrieval was done using a microwave. After incubation with freshly made 3% H_2_O_2_ for 10 min, the tissues were blocked in a blocking buffer for another 10 min at room temperature. Then the tissues were incubated with the primary antibodies, followed by secondary horseradish peroxidase (HRP) antibodies (Cell Signaling Technology) and an opal working solution (Akoya Biosciences). Primary antibodies recognizing the following antigens were used: MZT2A (1:20; Abcam), ALG3 (1:25; Abcam), GAPDH (1:1,500; Abcam), GPI (1:3,000; Abcam), and ATIC (1:200; Abcam). The slides were then mounted with ProLong Gold Antifade Reagent with DAPI and scanned using a confocal microscope (LEICA, Japan). Fluorescence images were acquired using a Vectra 2 intelligent slide analysis system using Vectra 2.0.8 (PerkinElmer). The mean fluorescence intensities (MFIs) of MZT2A, ALG3, GAPDH, GPI, and ATIC were measured.

### Immunohistochemistry of CD8+ T Cell

The distribution of CD8+ T cells in the lung cancer tissue array (HLugA180Su07) was evaluated by immunohistochemistry staining. The tissue array was incubated in a dry oven at 63°C for approximately 1 h, deparaffinized in xylene, and then rehydrated with graded ethanol solutions. After antigen retrieval, the array was incubated with a primary antibody against CD8 (DAKO, IR623) overnight at 4°C in a humidified chamber, followed by incubation at room temperature for 30 min with the secondary antibody (Envision+/HRP, Rabbit, DAKO). Subsequently, the tissue array was incubated with a 3,3′-diaminobenzidine (DAB) solution for 5 min. Finally, the tissue array was counterstained with hematoxylin. The immunostained slide was evaluated by two experienced pathologists blinded to clinicopathological characteristics, and the percentage of CD8 positive cells was annotated.

### Subgroup Analysis to Evaluate the Performance of the Model

To test the performance of the model, the risk score in different subgroups of age, sex, T stage, N stage, etc., was evaluated in TCGA dataset, GEO dataset, and the external validation dataset. Moreover, the Kaplan–Meier method and log-rank test were used to evaluate the performance of prognosis prediction in different subgroups. Differences in risk scores between different clinical characteristics were analyzed by GraphPad Prism 7.0. A Student’s t-test was used for comparison between two groups. ANOVA was used for comparison between three or more groups. p <0.05 was considered statistically significant.

## Results

### The Clinical Characteristics of The Cancer Genome Atlas Cohort

**Figure 1:**
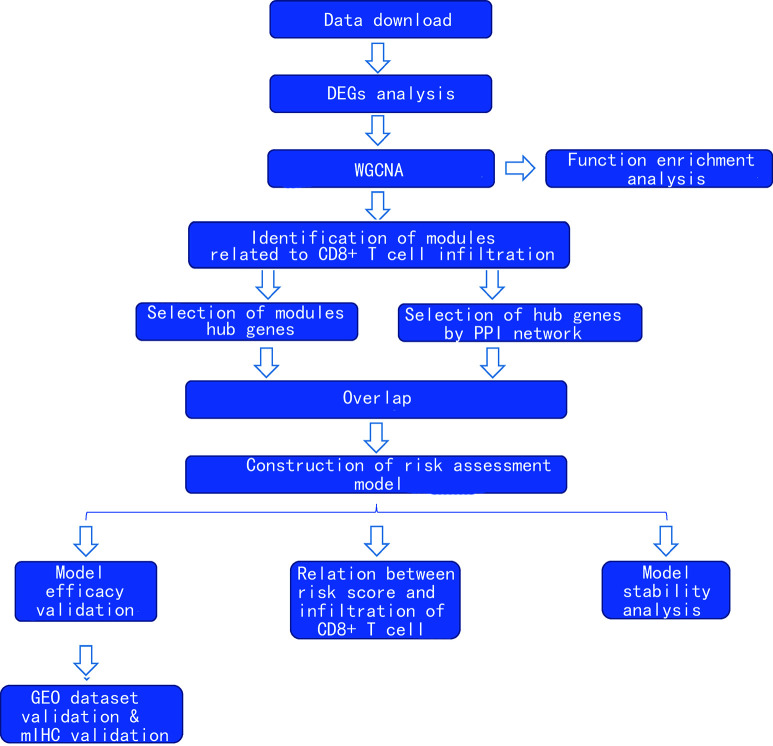
Flowchart of the study.

After exclusion of those cases with deficient clinical information, 529 cases were included in this study. Of these, 478 were cancer tissues and 51 were normal tissues. The clinical characteristics are shown in **Supplementary Table 1**. The flowchart of this study is shown in **Figure 1**.

### Identification of Differentially Expressed Genes and Construction of Gene Co-Expression Network

**Figure 2:**
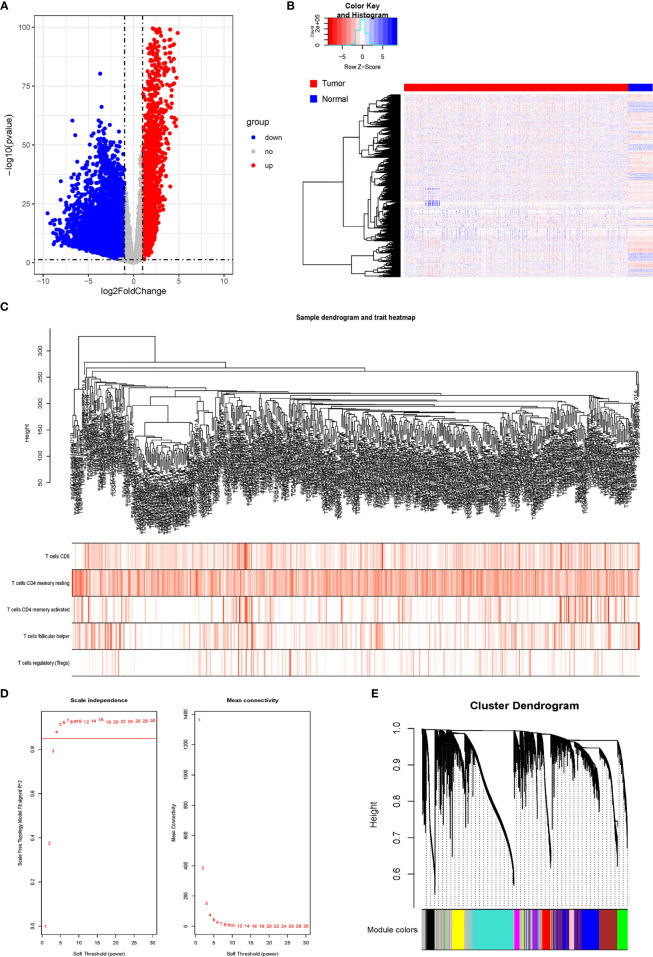
Analysis of DRGs and WGCNA. **(A)** Volcano plot of differential genes. **(B)** Heatmap of DEGs. **(C)** Sample clustering of WGCNA. **(D)** Screening with soft threshold. **(E)** Clustering of DEGs. DRG, differentially regulated gene; WGCNA, weighted gene co-expression network analysis; DEG, differentially expressed gene.

After comparing the expressions of LUAD tissues with those of normal tissues in TCGA-LUAD cohort, we identified 8,807 DEGs, including 2,172 upregulated genes and 6,635 downregulated genes (**Figures 2A, B**). The gene co-expression network was then constructed using the 8,807 DEGs. β = 4 (scale-free R_2_ > 0.85) was set as the soft-threshold power to build a scale-free network (**Figure 2D**). Furthermore, we used the dynamic hybrid cutting method to construct a hierarchical clustering tree. Each leaf on the tree represented an individual gene, and genes with similar expression data were gathered to form a tree branch representing a gene module. Eleven modules were generated (**Figures 2C, E**).

### Identification of Key Module Related to CD8+ T-Cell Infiltration

**Figure 3:**
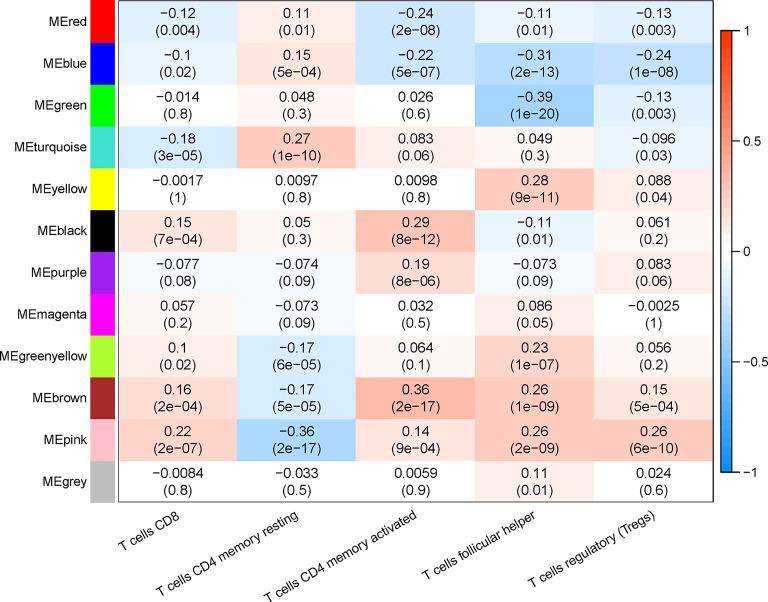
Correlation between infiltration of subtypes of T cells and different gene modules.

The proportion of immune cells in each sample was calculated based on gene expression by CIBERSORT. Seven T-cell subtypes were included: CD8+ T cells, CD4 naive T cells, CD4 memory resting T cells, CD4 memory activated T cells, follicular helper T cells, regulatory T cells (Tregs), and gamma delta T cells. Significantly, no CD4 naive T cells and gamma delta T cells were found. The proportion of each T cell subtype was extracted as the phenotype data, and its associations with the WGCNA modules were analyzed. The highest correlations were found between genes in the pink modules (275 genes) and CD8+ T cells (R^2^ = 0.22, p < 0.01). Hence, the genes in the pink modules were used in the subsequent analyses (**Figure 3**).

### Function Enrichment Analysis

**Figure 4:**
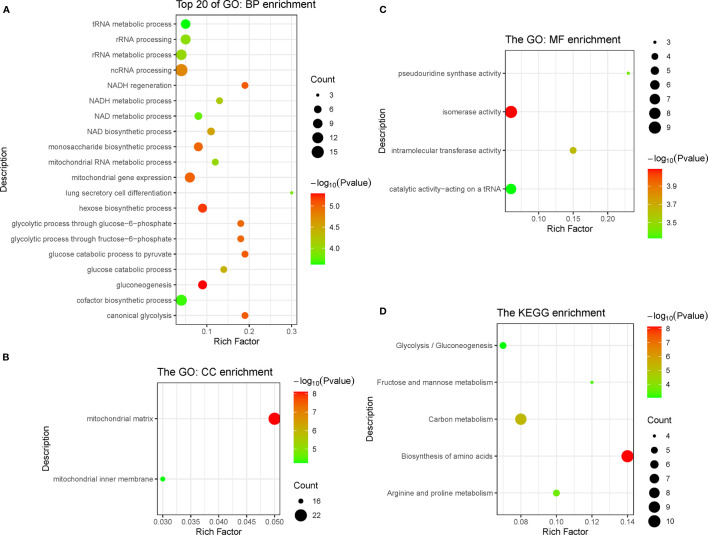
Function enrichment analysis of genes in pink module, including three types of GO enrichment analysis **(A–C)** and KEGG enrichment analysis **(D)**. GO, Gene Ontology; KEGG, Kyoto Encyclopedia of Genes and Genomes.

In the pink module, 275 genes were analyzed by Gene Ontology (GO) and Kyoto Encyclopedia of Genes and Genomes (KEGG) function enrichment analyses. GO analysis showed that the main enriched pathways were RNA metabolic processes, glucose metabolic processes, mitochondrial matrix, mitochondrial inner membrane, heterogeneous enzymatic activity, and tRNA catalytic activity (**Figures 4A–C**). The main pathways found enriched by KEGG were sugar metabolism and arginine/proline metabolism, and the enriched functions were mainly related to cell respiration (**Figure 4D**).

### Identification and Validation of Hub Genes Related to CD8+ T-Cell Infiltration

**Figure 5:**
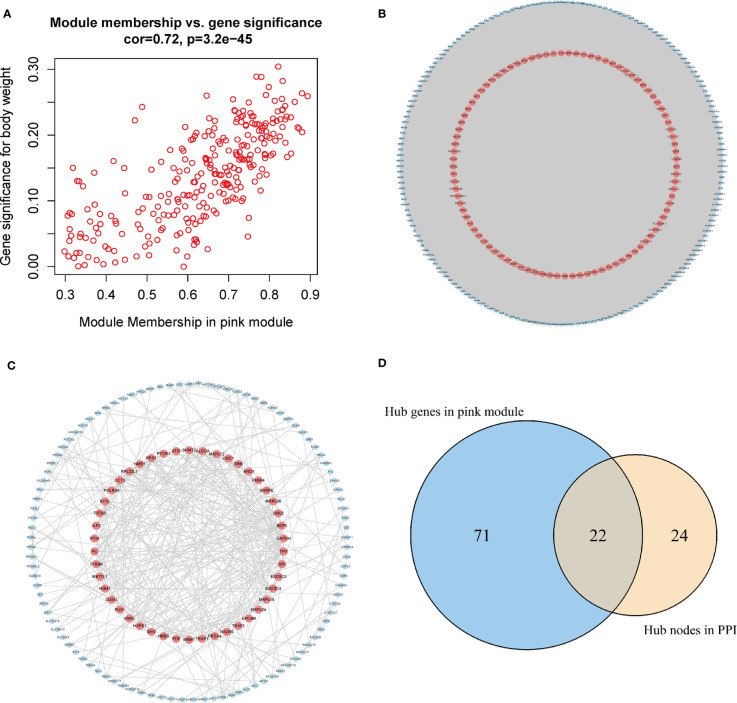
Acquisition of hub genes related to immune cell infiltration. **(A)** Correlation scatter diagram between pink genes and phenotypes of immune cell infiltration. **(B)** Pink gene co-expression network (red: the screened hub genes). **(C)** PPI network (red: the screened hub proteins). **(D)** Venn map from the two types of screening. PPI, protein–protein interaction.

The genes in the pink module were imported into Cytoscape to build a co-expression network (**Figure 5A**), and a total of 93 hub genes were obtained at degree >260 (**Figure 5B**). One hundred forty-eight interactions with the proteins encoded by the genes of the pink module were identified by the PPI network, and then 46 hub proteins were selected at the degree >5 (**Figure 5C**). By overlapping the genes in Cytoscape and the PPI network, we acquired 117 hub genes related to CD8+ T-cell infiltration (**Figure 5D**). Of them, VARS (originating from the PPI network) does not belong to the pink module or DEGs and thus was excluded from the subsequent analysis. Finally, 116 hub genes associated with CD8+ T-cell infiltration were obtained.

### Identification of Prognosis-Related Key Genes and Construction of a Risk Assessment Model

**Figure 6:**
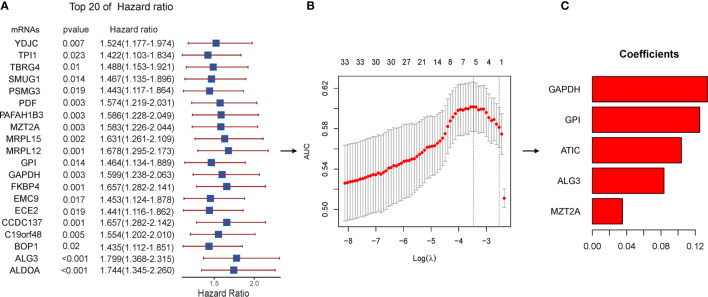
Lasso regression with the Cox single-factor regression results. **(A)** Top 20 genes of HR obtained from batched Cox single-factor regression. **(B)** Results of lambda screening. **(C)** Statistics of regression coefficients with the significantly related genes obtained from Lasso regression. Lasso, least absolute shrinkage and selection operator.

To identify prognosis-related genes from the hub genes, 116 genes were subjected to univariable Cox regression analysis. In total, 34 genes were found significantly associated with the prognosis (**Figure 6A**). We then used Lasso-penalized Cox regression analysis and identified five genes independently correlated with prognosis (**Figure 6B**). Based on the expressions and correlation coefficients of these five genes, a risk assessment model was established, where Risk Score = MZT2A * 0.035 + ALG3 * 0.084 + ATIC * 0.104 + GPI * 0.125 + GAPDH * 0.134 (**Figure 6C**).

### Validation of Risk Assessment Model

**Figure 7:**
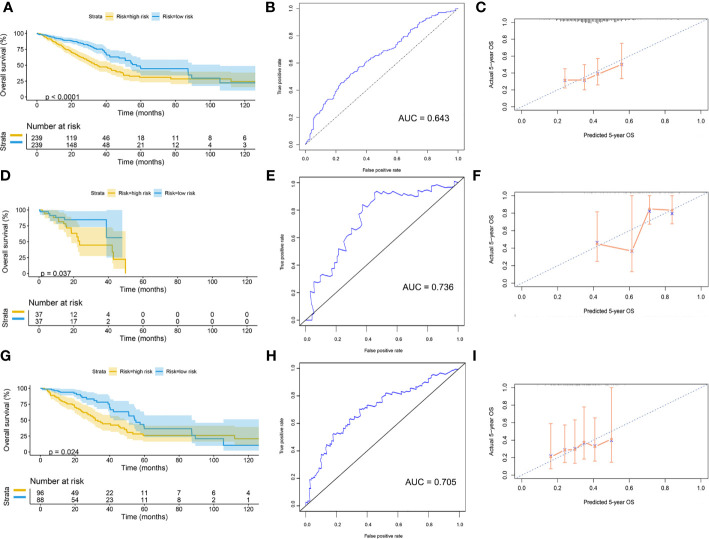
Validation of model by TCGA cohort. **(A)** Kaplan–Meier curve of overall cohort. **(B)** ROC curve of overall cohort. **(C)** Calibration curve of overall cohort. **(D)** Kaplan–Meier curve of mutation-type cohort. **(E)** ROC curve of mutation-type cohort. **(F)** Calibration curve of mutation-type cohort. **(G)** Kaplan–Meier curve of wild-type cohort. **(H)** ROC curve of wild-type cohort. **(I)** Calibration curve of wild-type cohort. TCGA, The Cancer Genome Atlas; ROC, receiver operating characteristic.

Patients were divided into high-risk and low-risk groups, with the median score at 6.53. The patients with the high-risk group showed a poorer 5-year overall survival (OS) compared with the patients in the low-risk group (low-risk *vs*. high-risk = 44.6% *vs*. 31.0%, p < 0.01) (**Figure 7A**). The ROC curves showed that the AUC of OS at 5-year was 0.643, which suggests that the prediction of the risk model has a good performance (**Figure 7B**). The calibration curve of the model suggested that the predicted 5-year OS closely correlated with the actual 5-year OS (**Figure 7C**). The subgroup analysis of the risk model suggested that the model had a good prediction performance in patients with wild-type EGFR status or mutation-type EGFR status (**Figures 7D–I**).

**Figure 8:**
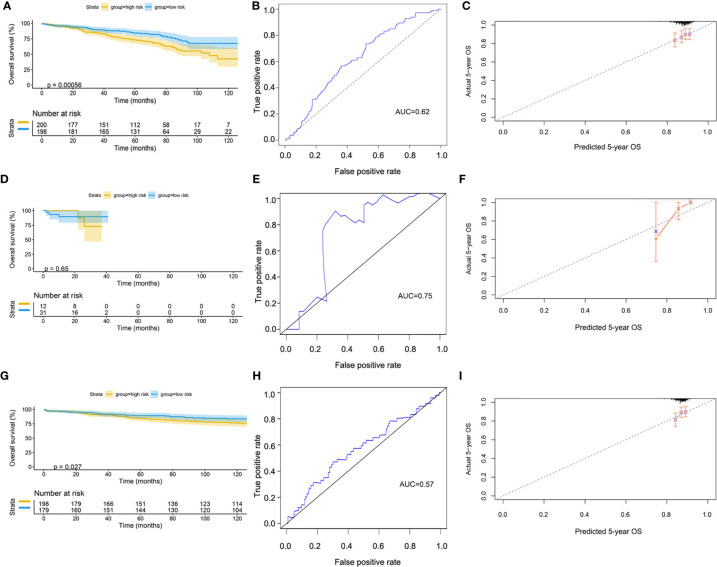
Validation of model by GEO cohort. **(A)** Kaplan–Meier curve of overall cohort. **(B)** ROC curve of overall cohort. **(C)** Calibration curve of overall cohort. **(D)** Kaplan–Meier curve of mutation-type cohort. **(E)** ROC curve of mutation-type cohort. **(F)** Calibration curve of mutation-type cohort. **(G)** Kaplan–Meier curve of wild-type cohort. **(H)** ROC curve of wild-type cohort. **(I)** Calibration curve of wild-type cohort. GEO, Gene Expression Omnibus; ROC, receiver operating characteristic.

The GSE72094 dataset was used to validate the risk model (**Supplementary Table 2**). After scoring, the cases were divided into high-risk and low-risk groups, with the median score at 5.34. The result of the Kaplan–Meier curve was similar to that of TCGA cohort (**Figure 8A**). Furthermore, the calibration curve, ROC curve, and the AUC (0.62) implied that this risk model had good prediction performance in the external dataset (**Figures 8B, C**). We also assessed the model in the patients with wild-type or mutation-type EGFR status. The results showed that the prognosis of the low-risk group did not have a significant difference from that of the high-risk group in the mutation-type cohort, which may be caused by the small sample size. However, the performance of the model in the wild-type cohort was good (**Figures 8D–I**).

**Figure 9:**
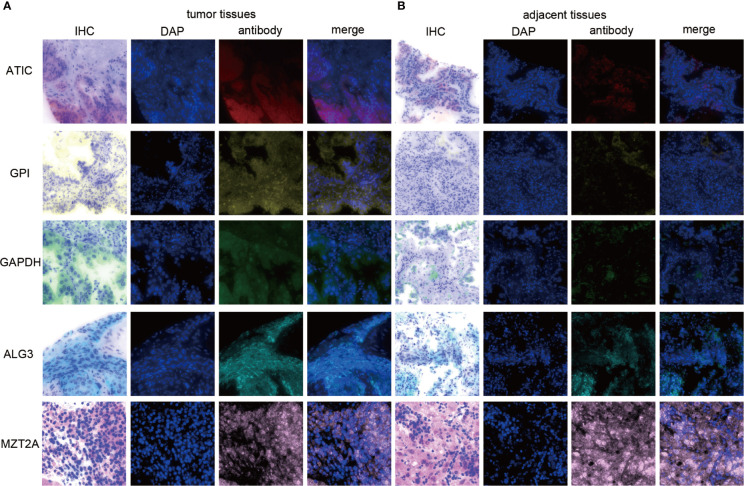
The expression of ATIC, GPI, GAPDH, ALG3, and MZT2A. **(A)** The expression of five proteins in tumor tissues. **(B)** The expression of five proteins in adjacent tissues.

**Figure 10:**
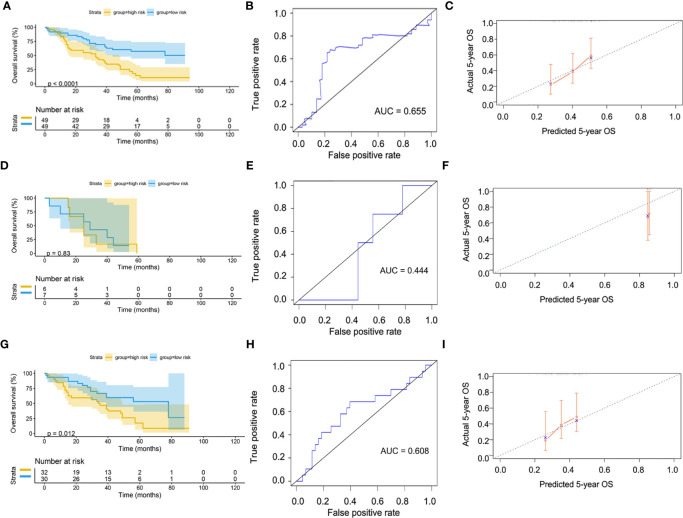
Validation of model by mIHC cohort. **(A)** Kaplan–Meier curve of overall cohort. **(B)** ROC curve of overall cohort. **(C)** Calibration curve of overall cohort. **(D)** Kaplan–Meier curve of mutation-type cohort. **(E)** ROC curve of mutation-type cohort. **(F)** Calibration curve of mutation-type cohort. **(G)** Kaplan–Meier curve of wild-type cohort. **(H)** ROC curve of wild-type cohort. **(I)** Calibration curve of wild-type cohort. ROC, receiver operating characteristic.

**Figure 11:**
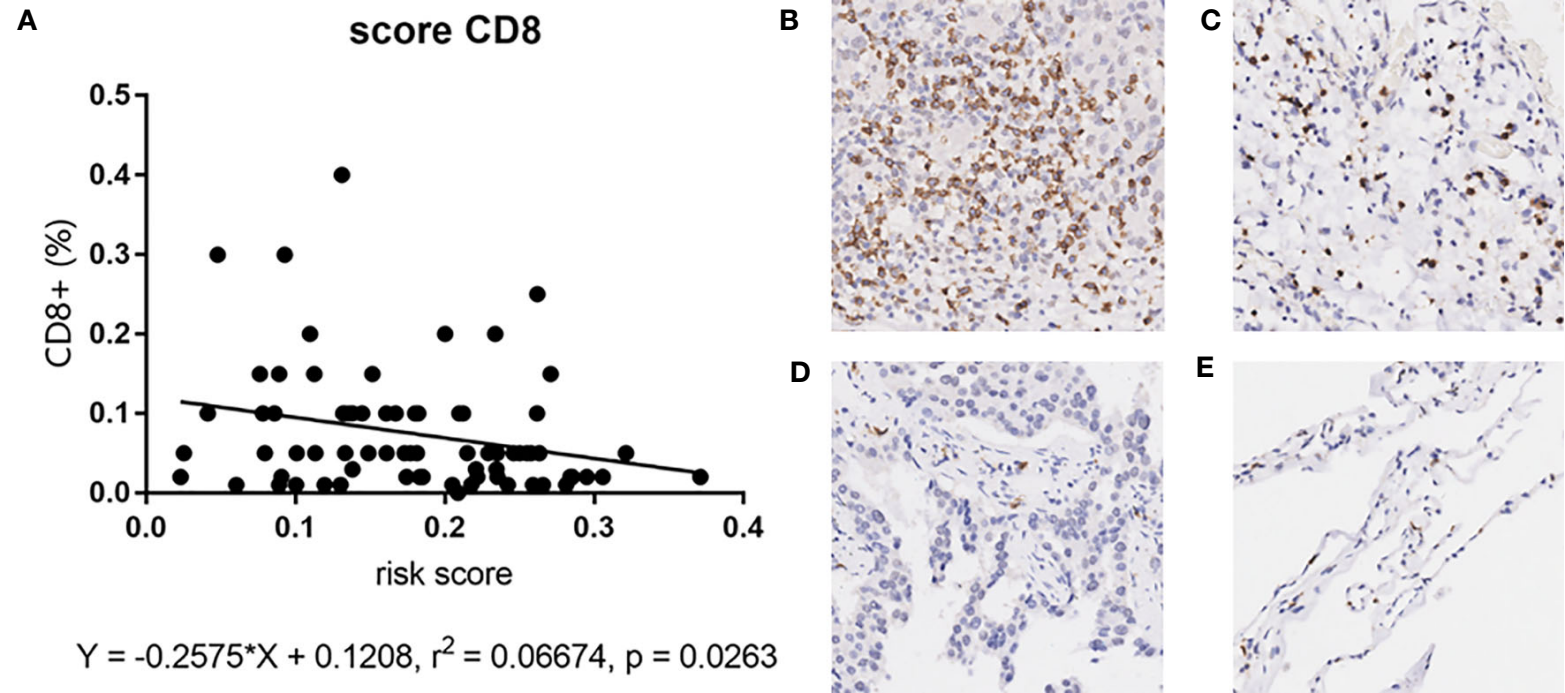
**(A)** The correlation between risk score and CD8+ T cell in mIHC cohort. **(B–E)** The distribution of CD8+ T cell in tumor tissues and adjacent tissues. High positive rate in tumor tissues **(A)** and adjacent tissues **(B)**. Low positive rate in tumor tissues **(C)** and adjacent tissues **(D)**.

Since our bioinformatics analysis was based on RNA sequences, we performed a multicolor immunofluorescence (mIHC) experiment on 98 LUAD tissues and 82 adjacent tissues from the protein perspective to validate the model. The follow-up time of the cohort was 1–94 months, and the median follow-up time was 39 months (interquartile range: 15–57). The median survival time was 50 months. The baseline characteristics of the cohort are shown in **Supplementary Table 3**. The expressions of five proteins were primarily located in the cytoplasm (**Figure 9**). We calculated the risk score based on fluorescence intensities and then divided the cohort into the low-risk group and high-risk group according to the median risk score. The results showed that the prognosis of the high-risk group was significantly poorer than that of the low-risk group (**Figure 10A**). The ROC curve, the calibration curve, and the AUC = 0.655 showed good performance (**Figures 10B, C**). In the subgroup analysis of EGFR status, the wild-type EGFR cohort showed similar results with the overall cohort. However, the mutation-type EGFR cohort results did not show significant differences between the prognosis of the high-risk group and the low-risk group. In addition, the performance of the risk model was poor, which may be caused by the small sample size (**Figures 10D–I**). We found that the risk score was significantly higher in the advanced T stage, N stage, and TNM stage. Furthermore, we found that the risk score was significantly negatively correlated to the infiltration of CD8+ T cells, which validated our bioinformatics analysis (**Figure 11A**). The distribution of CD8+ T cells is shown in **Figures 11B–E**.

### Correlation Analysis Between Genes in the TIMER Database and Subtypes of Immune Cells

**Figure 12:**
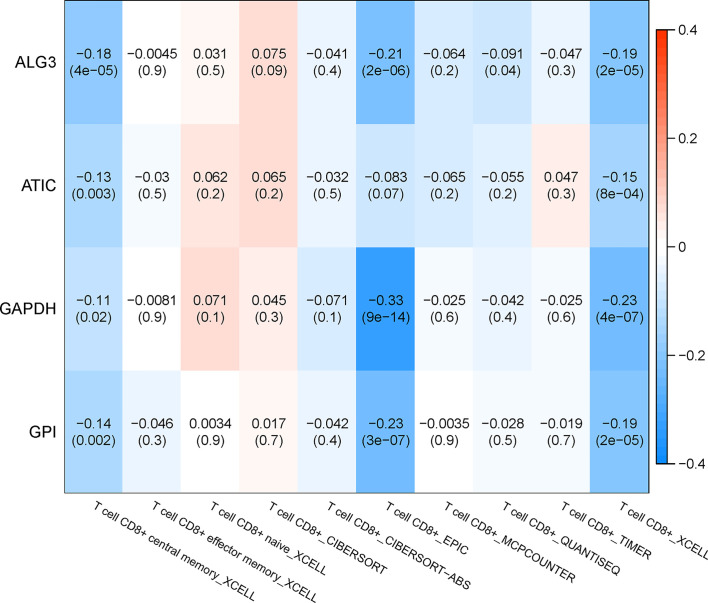
Correlation analysis between genes in the TIMER database and subtypes of immune cells.

The relationship between the key genes and CD8+ T cells was calculated online based on the TIMER database. Except for the lack of information on MZT2A, the remaining four genes were negatively correlated with CD8+ T cells significantly, according to both the XCELL algorithm and EPIC algorithm. Especially, the correlation coefficient of GAPDH with CD8+ T cells is −0.23 according to the XCELL algorithm (p < 0.01) and is −0.33 according to the EPIC algorithm (p < 0.01). These results implied that the expression of those genes might be negatively correlated with the infiltration of CD8+ T cells. The heatmap of key genes is shown in **Figure 12**.

### Subgroup Analysis

**Figure 13:**
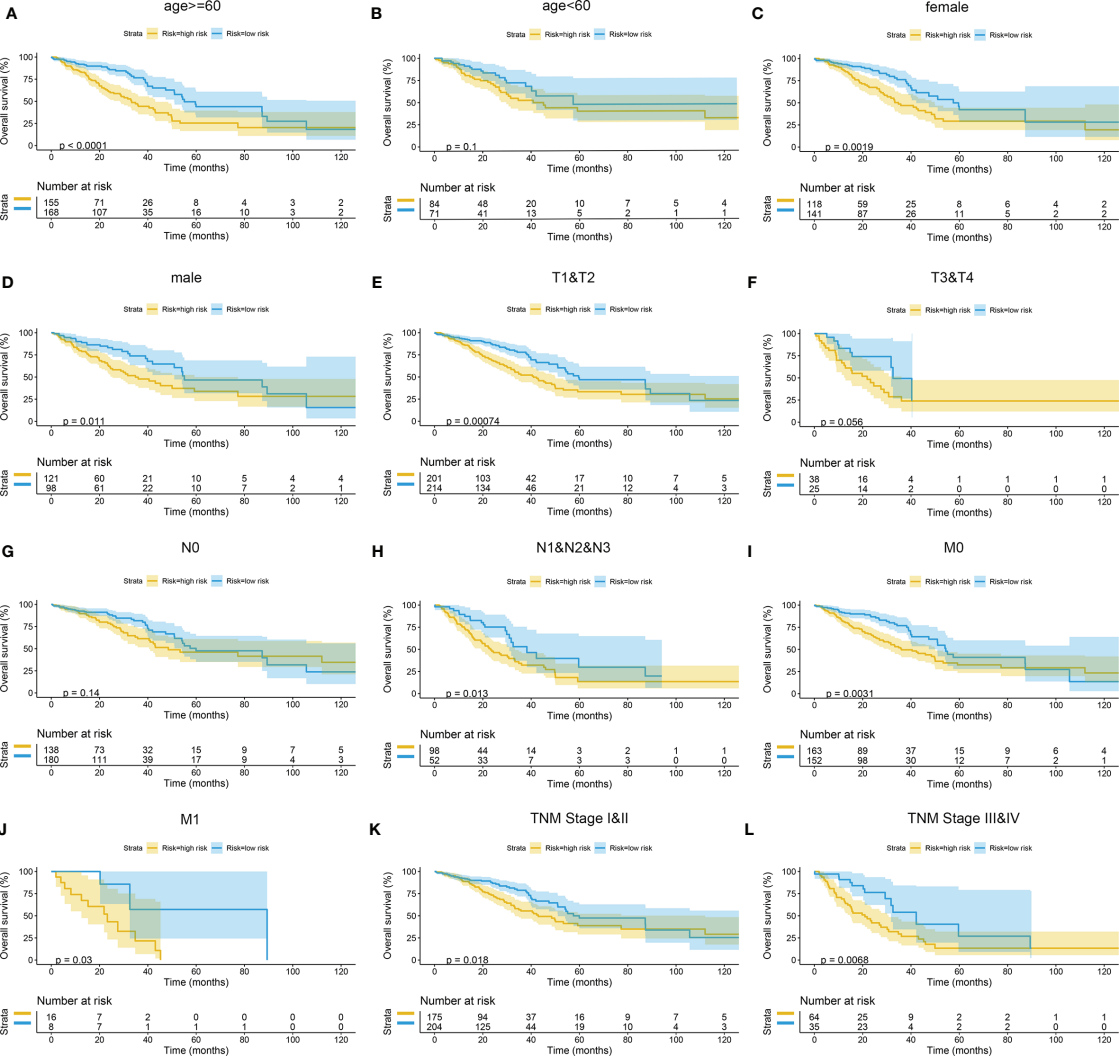
Subgroup survival analysis of TCGA cohort. **(A)** Age ≥ 60. **(B)** Age < 60. **(C)** Female. **(D)** Male. **(E)** Stages T1 and T2. **(F)** Stages T3 and T4. **(G)** Stage N0. **(H)** Stages N1, N2, and N3. **(I)** Stage M0. **(J)** Stage M1. **(K)** TNM stages I and II. **(L)** TNM stages III and IV. TCGA, The Cancer Genome Atlas.

**Figure 14:**
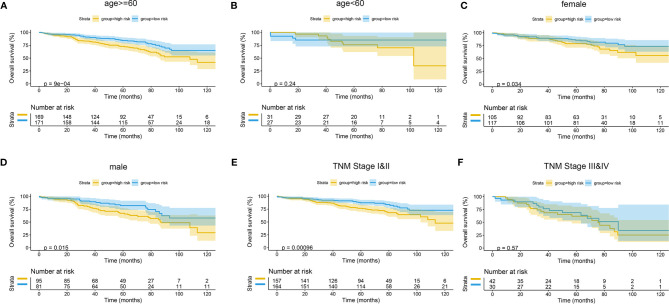
Subgroup survival analysis of GEO cohort. **(A)** Age ≥ 60. **(B)** Age < 60. **(C)** Female. **(D)** Male. **(E)** TNM stages I and II. **(F)** TNM stages III and IV. GEO, Gene Expression Omnibus.

**Figure 15:**
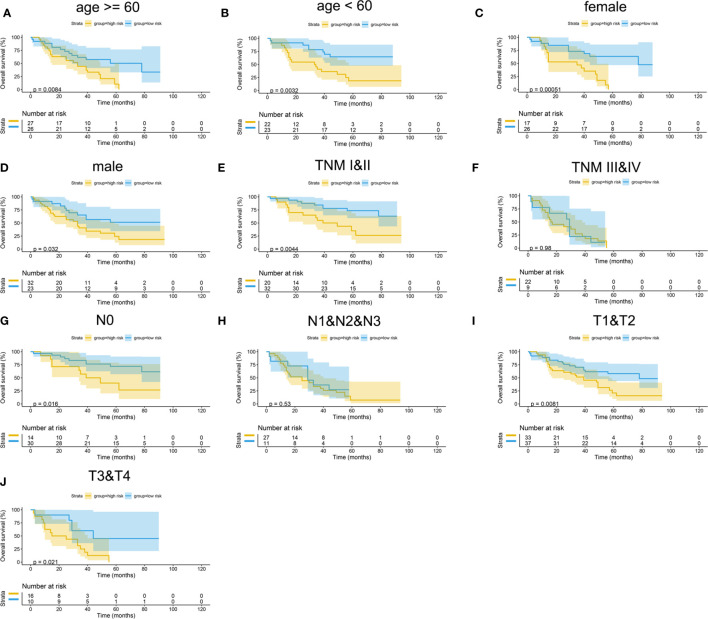
Subgroup survival analysis of mIHC cohort. **(A)** Age ≥ 60. **(B)** Age < 60. **(C)** Female. **(D)** Male. **(E)** TNM stages I and II. **(F)** TNM stages III and IV. **(G)** Stage N0. **(H)** Stages N1, N2, and N3. **(I)** Stages T1 and T2. **(J)** Stages T3 and T4.

The Kaplan–Meier curves were plotted in subgroups of data from TCGA database, including age, gender, T stage, N stage, M stage, and TNM stage. Although the prognosis of the high-risk group and the low-risk group was not significantly different in the age <60 group or the N0 group, the high-risk group had a poor prognosis in other subgroups (**Figure 13**). Similarly, the model underwent subgroup analysis with the GEO and mIHC cohorts, and the same results were found (**Figures 14**, **15**).

The differences of risk scores among age, gender, M stage, N stage, T stage, and TNM stage were tested in TCGA cohort. The results showed that the risk scores were higher in men, M1 stage, N2 or N3 stage, T3 or T4 stage, and TNM stage III or IV (**Supplementary Figure 1**). Similarly, differences in risk scores were detected in the GEO cohort and the mIHC cohort. The results showed that the advanced TNM stage had a higher score. Furthermore, the distribution of the risk scores between different EGFR statuses was tested in the GEO cohort, and the results showed that the risk scores were significantly higher in the wild type. However, in the mIHC cohort, we found that the risk score distribution in EGFR status was not significantly different. Hence, whether these five genes are related to EGFR status needs further exploration (**Supplementary Figures 2**, **3**).

## Discussion

LUAD is the most common type of lung cancer (24). Nowadays, surgery combined with chemotherapy, targeted therapy, or immunotherapy is the primary treatment strategy for most LUAD patients (25–29). For non-oncogene advanced LUAD, chemo-immunotherapy is an essential treatment strategy. In recent years, several reports found that the immune microenvironment plays a critical role in it. CD8+ T cells are the central effector cells of anti-tumor immunity (30–33). Identification of the key genes related to the infiltration of CD8+ T cells may offer new insights for research on the mechanism of tumor immunotherapy.

This work analyzed the expressions of 529 LUAD-related samples (478 cancer tissues and 51 paracancerous tissues) from TCGA database. As a result, 8,807 DEGs were identified, including 2,172 upregulated genes and 6,635 downregulated genes. We constructed a co-expression network by WGCNA and then identified the gene module most significantly correlated with CD8+ T cells combining with the CIBERSORT algorithm based on the DEGs. Subsequently, we identified five key genes (MZT2A, ALG3, ATIC, GPI, and GAPDH) related to prognosis and CD8+ T-cell infiltration through a co-expression network PPI network analysis and Lasso-penalized Cox regression analysis.

MZT2A (Mitotic Spindle Organizing Protein 2A) is a protein–encoding gene, but very little research is available on this gene. Recently, Wang et al. reported that MZT2A mRNA and protein levels were overexpressed in NSCLC and associated with poor NSCLC prognosis. Upregulation of MZT2A could promote NSCLC cell viability and invasion by overexpressing LGALS3BP *via* the MTZ2A MOZART2 domain and Akt phosphorylation (34). However, mechanisms on how MZT2A influences tumor prognosis and CD8+ T-cell infiltration should be further explored.

ALG3 (α-1,3-mannose glycosyl transferase) belongs to the ALG family and is located on the chromosomal region 3q27.1. ALG3 upregulation is related to lymph node metastasis of esophageal squamous cell carcinoma (35) and the proliferation of cervical cancer cells (36). ALG3 expression is higher in NSCLC tissues than in normal tissues and is associated with a higher T stage, lymph node metastasis, tissue differentiation, and prognosis (37). Similar to MZT2A, there are no reports on the relationship between ALG3 and CD8+ T-cell infiltration, which we will further explore.

ATIC (5-aminoimidazole-4-carboxamide ribonucleotide formyltransferase/IMP cyclohydrolase) encodes a bifunctional protein and catalyzes the last two steps in *de novo* synthesis of purines. ATIC is overexpressed in hepatic cell carcinoma and is associated with a poor prognosis in patients (38). The fused protein between ATIC and anaplasia lymphoma kinase (ALK, a common oncogene) was discovered in lymphoma patients (39, 40). Interestingly, frame-shift mutations and missense mutations of ATIC were found in a case of radiation sensitivity, and biochemical research showed that purine biosynthesis involving ATIC might help with DNA damage repair (41). Hence, these results imply that ATIC may be involved in tumorigenesis and may influence the survival of cancer cells. Additional research would be needed to explore the mechanism of ATIC and its relationship with CD8+ T-cell infiltration.

GPI (glucose-6-phosphate isomerase) is an enzyme that participates in the glycolysis pathway. GPI is a cytoplast dimer that can catalyze the conversion from glucose-6-phosphate to fructose-6-phosphate. GPI is a protein similar to the autocrine movement factors involved in the migration and invasion of tumor cells and angiogenesis (42). In various cancers, the expression of GPI is induced by c-Myc, and HIF-1 is overexpressed at the same time (43, 44). HIF-1 can induce GBE1 upregulation, which would decrease CCL5 and CXCL10 secretion, hindering the recruitment of CD8+ T lymphocytes (45, 46). GPI can also induce the protein expression of matrix metalloproteinase-3 and thereby promote the invasiveness of tumors (47). GPI, which is overexpressed in renal cancer, plays a role in tumor progression and is negatively correlated with the clinical prognosis of patients (48). However, the role of GPI in lung cancer has not been investigated to date.

GAPDH (glyceraldehyde-3-phosphate dehydrogenase) is one of the housekeeping proteins, and the mechanism of its anaerobic conversion to glucose critically regulates tissue regeneration and tumor growth (49, 50). Cancer cells can persistently survive under metabolic stress, anoxia, or starvation; and their glycolysis capacity must be improved by the Warburg effect, such as to improve the activity of enzymes involved in this function. Herein, the total glycolysis flux rate is precisely decided by the conversion stage from GALP (glyceraldehyde-3-phosphate) to biphosphoglyceride and is regulated by the activity of GAPDH (51). As an essential factor in the speed-limiting step of glycolysis, it plays a pivotal role in the energy metabolism of cancer cells. Hence, increased GAPDH activity will increase glycolysis rate and promote tumor growth, leading to poor prognosis (52). Anoxia is one of the major phenomena during tumor growth and activates the HIF-1α transcription factor to upregulate GAPDH expression (53, 54). In addition, upregulation of GAPDH may enhance HIF-1α transcription and activity, restricting the recruitment of CD8+ T lymphocytes (45, 46, 55). Moreover, the high activity of GAPDH increased the mobility of cancer cells, and epithelial–mesenchymal transition (EMT) markers are significantly associated. Colon cancer cell chromatin immune precipitation experiments proved the direct interaction between GAPDH and SPI transcripts, leading to the upregulation of the main regulatory factor in EMT, the zinc finger protein SNAI1 (Snail) (56–58). The initiation of GAPDH synthesis may be a protection mechanism for tumor cells to regulate metabolism and improve survival under anoxic conditions. Therefore, our results indicate that the expression of GAPDH may influence prognosis.

Based on these five genes, we established a risk score model. We found that the risk score could reasonably predict the prognosis of LUAD, and it was negatively related to the CD8+ T-cell infiltration and correlated with the advanced tumor stage. These results implied that these five genes might play a role in the infiltration of CD8+ T cells into the immune microenvironment. Among these genes, GAPDH and GPI may influence the infiltration of CD8+ T cells through the HIF-1/GBE1 pathway (45, 46, 55). Furthermore, the risk score was significantly upregulated in tumor tissues and correlated with advanced tumor stage. The validation of the overall cohort results by GEO dataset and the cohort of tissue microarray was consistent with the results of TCGA. Because the EGFR status is critical in LUAD, we also performed the subgroup analysis of the EGFR status. The performance of the risk model in both wild type and mutation type was good in TCGA cohort, as well as in the GEO cohort. However, the prediction accuracy is deficient in patients with mutation-type EGFR status in the mIHC cohort due to the small sample size. In a future study, we will increase the sample size of patients with mutation-type EGFR status to verify the risk model.

Nevertheless, this study also has some limitations. First, the mechanisms about how the genes affect the infiltration of CD8+ T cells were not explored in this report, but they will be investigated in a future study. Secondly, we constructed a risk score model that depended on gene expression but did not consider gene mutation, methylation, or other genetic events that can affect the occurrence and progression of cancers. In a subsequent study, we may consider more genetic modification to make our risk model more accurate. Finally, a large-sample prospective study is needed to further validate the clinical applicability of the risk score.

## Conclusions

In conclusion, this study may provide a novel risk assessment model for prognosis prediction and a new prospect for exploring the mechanism of tumor immune microenvironment related to CD8+ T-cell infiltration in LUAD.

## Data Availability Statement

The original contributions presented in the study are included in the article/**Supplementary Material**. Further inquiries can be directed to the corresponding author.

## Ethics Statement

The study was approved by the ethics committee of National Cancer Center, Cancer Hospital, Chinese Academy of Medical Sciences and Peking Union Medical College, and all enrolled patients had given written informed consent to participate in this study.

## Author Contributions

MD: conceptualization (lead), formal analysis (Lead), methodology (lead), and writing—original draft (lead). YL: methodology (equal), project administration (equal), supervision (equal), validation (equal), and writing—original draft (lead). ZL: data curation (equal), resources (equal), and writing—original draft (supporting). XL: validation (equal) and writing—original draft (supporting). ML: validation (equal) and writing—original draft (supporting). BZ: supervision (supporting). YG: funding acquisition (lead), project administration (lead), and writing—review and editing (lead). All authors contributed to the article and approved the submitted version.

## Funding

This work was supported by the National Key R&D Program of China (Grant No. 2020YFE0202200).

## Conflict of Interest

The authors declare that the research was conducted in the absence of any commercial or financial relationships that could be construed as a potential conflict of interest.

## Publisher’s Note

All claims expressed in this article are solely those of the authors and do not necessarily represent those of their affiliated organizations, or those of the publisher, the editors and the reviewers. Any product that may be evaluated in this article, or claim that may be made by its manufacturer, is not guaranteed or endorsed by the publisher.

## Supplementary Material

Supplementary Figure 1 Differences of risk score in subgroup of TCGA cohort. **(A)** age; **(B)** T stage; **(C)** N stage; **(D)** M stage; **(E)** sex; **(F)** TNM stage.

Supplementary Figure 2 Differences of risk score in subgroup of GEO cohort. **(A)** sex; **(B)** age; **(C)** TNM stage; **(D)** EGFR status.

Supplementary Figure 3 Differences of risk score in subgroup of mIHC cohort. **(A)** sex; **(B)** age; **(C)** T stage; **(D)** N stage; **(E)** EGFR status; **(F)** ALK status; **(G)** TNM stage.

## References

[1] Siegel R Miller K Fuchs H Jemal A. Cancer Statistics, 2021. CA Cancer J Clin (2021) 71(1):7–33. doi: 10.3322/caac.21654 33433946

[2] Noguchi M Morikawa A Kawasaki M Matsuno Y Yamada T Hirohashi S. Small Adenocarcinoma of the Lung. Histologic Characteristics and Prognosis. Cancer (1995) 75(12):2844–52. doi: 10.1002/1097-0142(19950615)75:12<2844::aid-cncr2820751209>3.0.co;2-# 7773933

[3] Zappa C Mousa S. Non-Small Cell Lung Cancer: Current Treatment and Future Advances. Transl Lung Cancer Res (2016) 5(3):288–300. doi: 10.21037/tlcr.2016.06.07 27413711 PMC4931124

[4] Sacher A Gandhi L. Biomarkers for the Clinical Use of PD-1/PD-L1 Inhibitors in Non-Small-Cell Lung Cancer: A Review. JAMA Oncol (2016) 2(9):1217–22. doi: 10.1001/jamaoncol.2016.0639 27310809

[5] Cai L Sun Y Wang K Guan W Yue J Li J. The Better Survival of MSI Subtype Is Associated With the Oxidative Stress Related Pathways in Gastric Cancer. Front Oncol (2020) 10:1269. doi: 10.3389/fonc.2020.01269 32850385 PMC7399340

[6] Chen D Mellman IJN. Elements of Cancer Immunity and the Cancer-Immune Set Point. Nature (2017) 541:321–30. doi: 10.1038/nature21349 28102259

[7] Tian Y Zhai X Yan W Zhu H Yu J. Clinical Outcomes of Immune Checkpoint Blockades and the Underlying Immune Escape Mechanisms in Squamous and Adenocarcinoma NSCLC. Cancer Med (2021) 10:3–14. doi: 10.1002/cam4.3590 33230935 PMC7826453

[8] Augustin R Delgoffe G Najjar YJC. Characteristics of the Tumor Microenvironment That Influence Immune Cell Functions: Hypoxia, Oxidative Stress, Metabolic Alterations. Cancers (Basel) (2020) 12:3802. doi: 10.3390/cancers12123802 PMC7765870 33348579

[9] Galon J Costes A Sanchez-Cabo F Kirilovsky A Mlecnik B Lagorce-Pagès C. Type, Density, and Location of Immune Cells Within Human Colorectal Tumors Predict Clinical Outcome. Science (2006) 313:1960–4. doi: 10.1126/science.1129139 17008531

[10] Mahmoud S Paish E Powe D Macmillan R Grainge M Lee A. Tumor-Infiltrating CD8+ Lymphocytes Predict Clinical Outcome in Breast Cancer. J Clin Oncol (2011) 29:1949–55. doi: 10.1200/JCO.2010.30.5037 21483002

[11] Sharma P Shen Y Wen S Yamada S Jungbluth A Gnjatic S. CD8 Tumor-Infiltrating Lymphocytes Are Predictive of Survival in Muscle-Invasive Urothelial Carcinoma. Proc Natl Acad Sci USA (2007) 104:3967–72. doi: 10.1073/pnas.0611618104 PMC1820692 17360461

[12] Donnem T Hald S Paulsen E Richardsen E Al-Saad S Kilvaer T. Stromal CD8+ T-Cell Density—A Promising Supplement to TNM Staging in Non-Small Cell Lung Cancer. Clin Cancer Res (2015) 21:2635–43. doi: 10.1158/1078-0432.Ccr-14-1905 25680376

[13] Al-Shibli K Donnem T Al-Saad S Persson M Bremnes R Busund L. Prognostic Effect of Epithelial and Stromal Lymphocyte Infiltration in Non-Small Cell Lung Cancer. Clin Cancer Res (2008) 14:5220–7. doi: 10.1158/1078-0432.CCR-08-0133 18698040

[14] Fu X Xu M Zhang H Li Y Li Y Zhang C. Staphylococcal Enterotoxin C2 Mutant-Directed Fatty Acid and Mitochondrial Energy Metabolic Programs Regulate CD8 T Cell Activation. J Immunol (2020) 205:2066–76. doi: 10.4049/jimmunol.2000538 32938730

[15] Lin T Gu J Qu K Zhang X Ma X Miao R. A New Risk Score Based on Twelve Hepatocellular Carcinoma-Specific Gene Expression Can Predict the Patients’ Prognosis. Aging (Albany NY) (2018) 10:2480–97. doi: 10.18632/aging.101563 PMC6188480 30243023

[16] Langfelder P Horvath S. WGCNA: An R Package for Weighted Correlation Network Analysis. BMC Bioinf (2008) 9:559. doi: 10.1186/1471-2105-9-559 PMC2631488 19114008

[17] Niemira M Collin F Szalkowska A Bielska A Chwialkowska K Reszec J. Molecular Signature of Subtypes of Non-Small-Cell Lung Cancer by Large-Scale Transcriptional Profiling: Identification of Key Modules and Genes by Weighted Gene Co-Expression Network Analysis (WGCNA). Cancers (Basel) (2019) 12:37. doi: 10.3390/cancers12010037 PMC7017323 31877723

[18] Liu H Sun Y Tian H Xiao X Zhang J Wang Y. Characterization of Long Non-Coding RNA and Messenger RNA Profiles in Laryngeal Cancer by Weighted Gene Co-Expression Network Analysis. Aging (Albany NY) (2019) 11:10074–99. doi: 10.18632/aging.102419 PMC6914418 31739287

[19] Newman A Liu C Green M Gentles A Feng W Xu Y. Robust Enumeration of Cell Subsets From Tissue Expression Profiles. Nat Methods (2015) 12:453–7. doi: 10.1038/nmeth.3337 PMC4739640 25822800

[20] Zhao S Lehrer J Chang S Das R Erho N Liu Y. The Immune Landscape of Prostate Cancer and Nomination of PD-L2 as a Potential Therapeutic Target. J Natl Cancer Inst (2019) 111:301–10. doi: 10.1093/jnci/djy141 30321406

[21] Zhang S Zhang E Long J Hu Z Peng J Liu L. Immune Infiltration in Renal Cell Carcinoma. Cancer Sci (2019) 110:1564–72. doi: 10.1111/cas.13996 PMC6501001 30861269

[22] Galvano A Gristina V Malapelle U Pisapia P Pepe F Barraco N. The Prognostic Impact of Tumor Mutational Burden (TMB) in the First-Line Management of Advanced Non-Oncogene Addicted Non-Small-Cell Lung Cancer (NSCLC): A Systematic Review and Meta-Analysis of Randomized Controlled Trials. ESMO Open (2021) 6:100124. doi: 10.1016/j.esmoop.2021.100124 33940346 PMC8111593

[23] Pepe F Pisapia P Gristina V Rocco D Micheli M Micheli P. Tumor Mutational Burden on Cytological Samples: A Pilot Study. Cancer Cytopathol (2021) 129:460–7. doi: 10.1002/cncy.22400 33378102

[24] Travis W Brambilla E Noguchi M Nicholson A Geisinger K Yatabe Y. International Association for the Study of Lung Cancer/American Thoracic Society/European Respiratory Society International Multidisciplinary Classification of Lung Adenocarcinoma. Thorac Oncol (2011) 6:244–85. doi: 10.1097/JTO.0b013e318206a221 PMC4513953 21252716

[25] Oser M Niederst M Sequist L Engelman JA. Transformation From Non-Small-Cell Lung Cancer to Small-Cell Lung Cancer: Molecular Drivers and Cells of Origin. Lancet Oncol (2015) 16:e165–72. doi: 10.1016/S1470-2045(14)71180-5 PMC4470698 25846096

[26] Blandin Knight S Crosbie P Balata H Chudziak J Hussell T Dive C. Progress and Prospects of Early Detection in Lung Cancer. Open Biol (2017) 7:170070. doi: 10.1098/rsob.170070 28878044 PMC5627048

[27] Deng W Xu T Xu Y Wang Y Liu X Zhao Y. Survival Patterns for Patients With Resected N2 Non-Small Cell Lung Cancer and Postoperative Radiotherapy: A Prognostic Scoring Model and Heat Map Approach. J Thorac Oncol (2018) 13:1968–74. doi: 10.1016/j.jtho.2018.08.2021 PMC6552655 30194035

[28] Herskovic A Mauer E Christos P Nagar H. Role of Postoperative Radiotherapy in Pathologic Stage IIIA (N2) Non-Small Cell Lung Cancer in a Prospective Nationwide Oncology Outcomes Database. J Thorac Oncol (2017) 12:302–13. doi: 10.1016/j.jtho.2016.09.135 27746190

[29] Kris M Gaspar L Chaft J Kennedy E Azzoli C Ellis P. Adjuvant Systemic Therapy and Adjuvant Radiation Therapy for Stage I to IIIA Completely Resected Non-Small-Cell Lung Cancers: American Society of Clinical Oncology/Cancer Care Ontario Clinical Practice Guideline Update. J Clin Oncol (2017) 35:2960–74. doi: 10.1200/JCO.2017.72.4401 28437162

[30] Adams N Grassmann S Sun JC. Clonal Expansion of Innate and Adaptive Lymphocytes. Nat Rev Immunol (2020) 20:694–707. doi: 10.1038/s41577-020-0307-4 32424244 PMC13119617

[31] van der Leun A Thommen D Schumacher TN. CD8 T Cell States in Human Cancer: Insights From Single-Cell Analysis. Nat Rev Cancer (2020) 20(4):218–32. doi: 10.1038/s41568-019-0235-4 PMC7115982 32024970

[32] Butterfield LJB. Cancer Vaccines. BMJ (2015) 350:h988. doi: 10.1136/bmj.h988 25904595 PMC4707521

[33] Appay V Douek D Price DA. CD8+ T Cell Efficacy in Vaccination and Disease. Nat Med (2008) 14:623–8. doi: 10.1038/nm.f.1774 18535580

[34] Wang H Jiang X Cheng Y Ren H Hu Y Zhang Y. MZT2A Promotes NSCLC Viability and Invasion by Increasing Akt Phosphorylation *via* the MOZART2 Domain. Cancer Sci (2021) 112:2210–22. doi: 10.1111/cas.14900 PMC8177791 33754417

[35] Shi Z Jiang Y Hao J Zhang Y Zhang T Shang L. Identification of Putative Target Genes for Amplification Within 11q13.2 and 3q27.1 in Esophageal Squamous Cell Carcinoma. Clin Transl Oncol (2014) 16:606–15. doi: 10.1007/s12094-013-1124-z 24203761

[36] Choi Y Bae S Kim Y Lee H Kim Y Park T. Gene Expression Profiles in Squamous Cell Cervical Carcinoma Using Array-Based Comparative Genomic Hybridization Analysis. Int J Gynecol Cancer (2007) 17:687–96. doi: 10.1111/j.1525-1438.2007.00834.x 17504382

[37] Ke S Qiu H Chen J Shi W Han C Gong Y. ALG3 Contributes to the Malignancy of Non-Small Cell Lung Cancer and Is Negatively Regulated by Mir-98-5p. Pathol Res Pract (2020) 216:152761. doi: 10.1016/j.prp.2019.152761 31899049

[38] Li M Jin C Xu M Zhou L Li D Yin YJC. Bifunctional Enzyme ATIC Promotes Propagation of Hepatocellular Carcinoma by Regulating AMPK-Mtor-S6 K1 Signaling. Cell Commun Signal (2017) 15:52. doi: 10.1186/s12964-017-0208-8 29246230 PMC5732395

[39] Trinei M Lanfrancone L Campo E Pulford K Mason D Pelicci P. A New Variant Anaplastic Lymphoma Kinase (ALK)-Fusion Protein (ATIC-ALK) in a Case of ALK-Positive Anaplastic Large Cell Lymphoma. Cancer Res (2000) 60:793–8. 10706082

[40] van der Krogt J Bempt M Ferreiro J Mentens N Jacobs K Pluys U. Anaplastic Lymphoma Kinase-Positive Anaplastic Large Cell Lymphoma With the Variant RNF213-, ATIC- and TPM3-ALK Fusions Is Characterized by Copy Number Gain of the Rearranged ALK Gene. Haematologica (2017) 102:1605–16. doi: 10.3324/haematol.2016.146571 PMC5685221 28659337

[41] Liu X Paila U Teraoka S Wright J Huang X Quinlan A. Identification of ATIC as a Novel Target for Chemoradiosensitization. Int J Radiat Oncol Biol Phys (2018) 100:162–73. doi: 10.1016/j.ijrobp.2017.08.033 PMC5736427 29029884

[42] Funasaka T Yanagawa T Hogan V Raz A. Regulation of Phosphoglucose Isomerase/Autocrine Motility Factor Expression by Hypoxia. FASEB J (2005) 19:1422–30. doi: 10.1096/fj.05-3699com 16126909

[43] Semenza GL. HIF-1 Mediates Metabolic Responses to Intratumoral Hypoxia and Oncogenic Mutations. J Clin Invest (2013) 123:3664–71. doi: 10.1172/JCI67230 PMC3754249 23999440

[44] Pusapati R Daemen A Wilson C Sandoval W Gao M Haley B. Mtorc1-Dependent Metabolic Reprogramming Underlies Escape From Glycolysis Addiction in Cancer Cells. Cancer Cell (2016) 29:548–62. doi: 10.1016/j.ccell.2016.02.018 27052953

[45] Li L Yang L Fan Z Xue W Shen Z Yuan Y. Hypoxia-Induced GBE1 Expression Promotes Tumor Progression Through Metabolic Reprogramming in Lung Adenocarcinoma. Signal Transduct Target Ther (2020) 5:54. doi: 10.1038/s41392-020-0152-8 32439898 PMC7242448

[46] Li L Yang L Cheng S Fan Z Shen Z Xue W. Lung Adenocarcinoma-Intrinsic GBE1 Signaling Inhibits Anti-Tumor Immunity. Mol Cancer (2019) 18:108. doi: 10.1186/s12943-019-1027-x 31221150 PMC6585057

[47] Itoh Y. Membrane-Type Matrix Metalloproteinases: Their Functions and Regulations. Matrix Biol (2015) 44–46:207–23. doi: 10.1016/j.matbio.2015.03.004 25794647

[48] Lucarelli G Rutigliano M Sanguedolce F Galleggiante V Giglio A Cagiano S. Increased Expression of the Autocrine Motility Factor Is Associated With Poor Prognosis in Patients With Clear Cell-Renal Cell Carcinoma. Medicine (Baltimore) (2015) 94:e2117. doi: 10.1097/MD.0000000000002117 26579829 PMC4652838

[49] Yang J Ren B Yang G Wang H Chen G You L. The Enhancement of Glycolysis Regulates Pancreatic Cancer Metastasis. Cell Mol Life Sci (2020) 77:305–21. doi: 10.1007/s00018-019-03278-z PMC11104916 31432232

[50] Woolbright B Ayres M Taylor JA. Metabolic Changes in Bladder Cancer. Urol Oncol (2018) 36:327–37. doi: 10.1016/j.urolonc.2018.04.010 29773495

[51] Shestov A Liu X Ser Z Cluntun A Hung Y Huang L. Quantitative Determinants of Aerobic Glycolysis Identify Flux Through the Enzyme GAPDH as a Limiting Step. Elife (2014) 9(3):e03342. doi: 10.7554/eLife.03342 PMC4118620 25009227

[52] Brzozowa-Zasada M Kurek J Piecuch A Stęplewska KJP. Correlation Study of GAPDH, Bcl-2, and Bax Protein Immunoexpression in Patients With Colorectal Adenocarcinoma. Prz Gastroenterol (2018) 13:322–31. doi: 10.5114/pg.2018.79813 PMC6300847 30581507

[53] Mikuriya K Kuramitsu Y Ryozawa S Fujimoto M Mori S Oka M. Expression of Glycolytic Enzymes Is Increased in Pancreatic Cancerous Tissues as Evidenced by Proteomic Profiling by Two-Dimensional Electrophoresis and Liquid Chromatography-Mass Spectrometry/Mass Spectrometry. Int J Oncol (2007) 30:849–55. doi: 10.3892/ijo.30.4.849 17332923

[54] Higashimura Y Nakajima Y Yamaji R Harada N Shibasaki F Nakano Y. Up-Regulation of Glyceraldehyde-3-Phosphate Dehydrogenase Gene Expression by HIF-1 Activity Depending on Sp1 in Hypoxic Breast Cancer Cells. Arch Biochem Biophys (2011) 509:1–8. doi: 10.1016/j.abb.2011.02.011 21338575

[55] Chiche J Pommier S Beneteau M Mondragón L Meynet O Zunino B. GAPDH Enhances the Aggressiveness and the Vascularization of Non-Hodgkin’s B Lymphomas *via* NF-Kb-Dependent Induction of HIF-1α. Leukemia (2015) 29:1163–76. doi: 10.1038/leu.2014.324 25394713

[56] Schneider M Knuesting J Birkholz O Heinisch J Scheibe RJB. Cytosolic GAPDH as a Redox-Dependent Regulator of Energy Metabolism. BMC Plant Biol (2018) 18:184. doi: 10.1186/s12870-018-1390-6 30189844 PMC6127989

[57] Liu K Tang Z Huang A Chen P Liu P Yang J. Glyceraldehyde-3-Phosphate Dehydrogenase Promotes Cancer Growth and Metastasis Through Upregulation of SNAIL Expression. Int J Oncol (2017) 50:252–62. doi: 10.3892/ijo.2016.3774 27878251

[58] Hao L Zhou X Liu S Sun M Song Y Du S. Elevated GAPDH Expression Is Associated With the Proliferation and Invasion of Lung and Esophageal Squamous Cell Carcinomas. Proteomics (2015) 15:3087–100. doi: 10.1002/pmic.201400577 25944651

