# Supplementary figures and images for: Identification of Key Genes Related to CD8+ T-Cell Infiltration as Prognostic Biomarkers for Lung Adenocarcinoma
Source: Front Oncol. 2021 Sep 28;11:693353. doi: 10.3389/fonc.2021.693353 (PMC8505972; doi:10.3389/fonc.2021.693353)

A

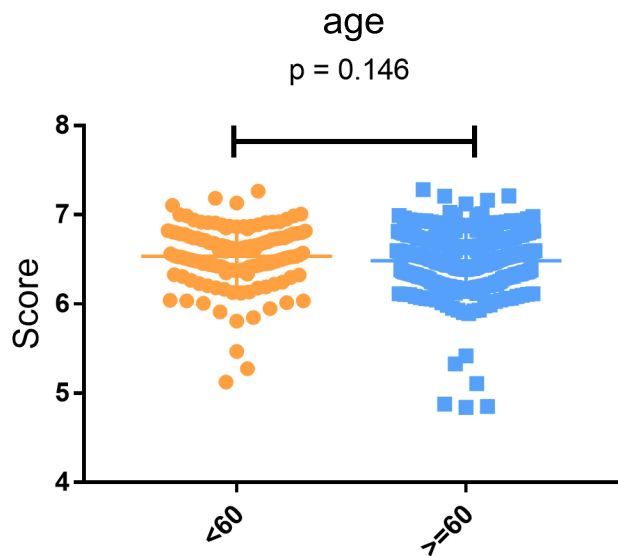

B

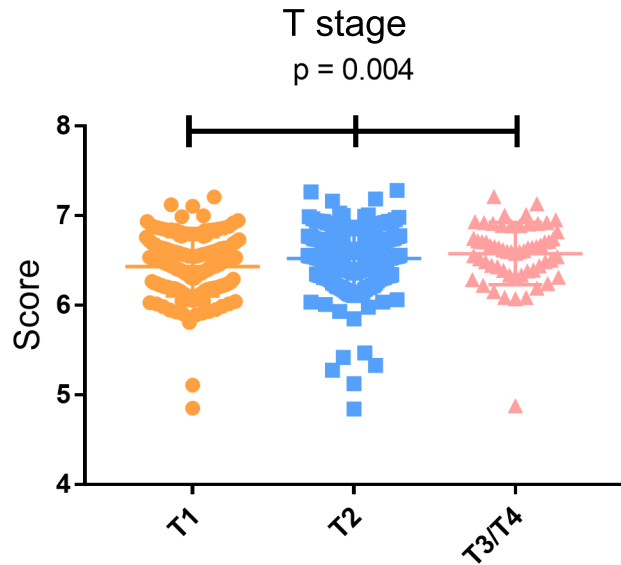

C

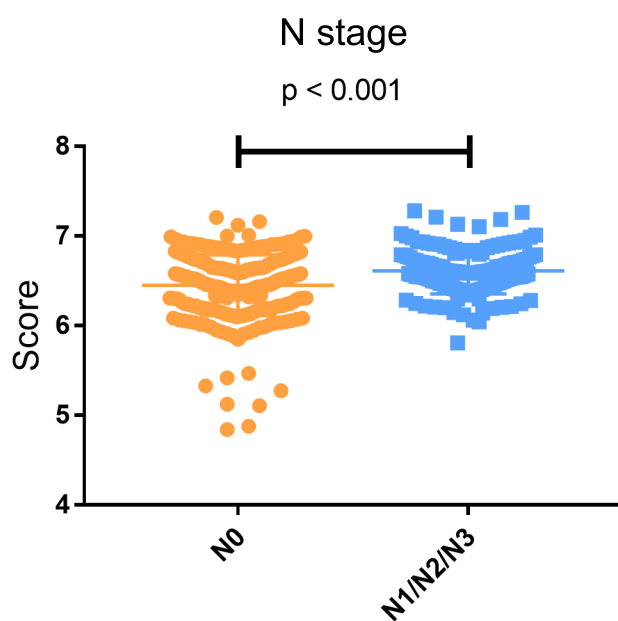

D

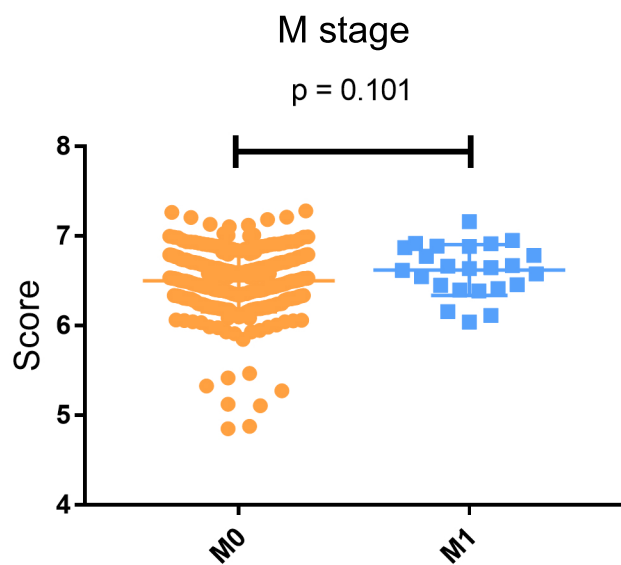

E

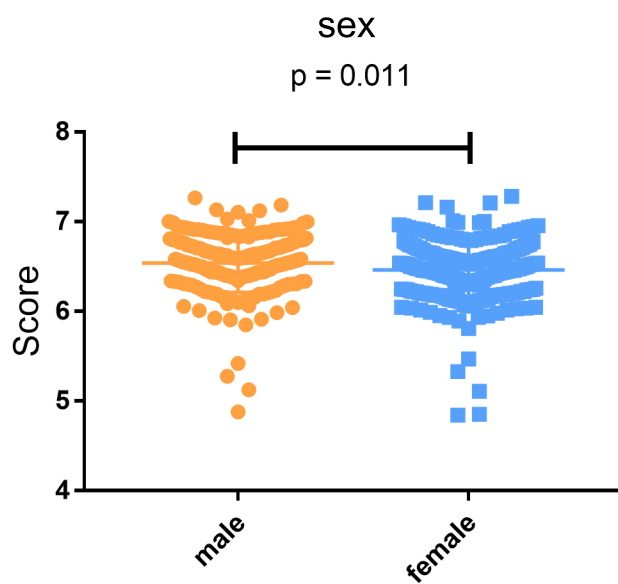

F

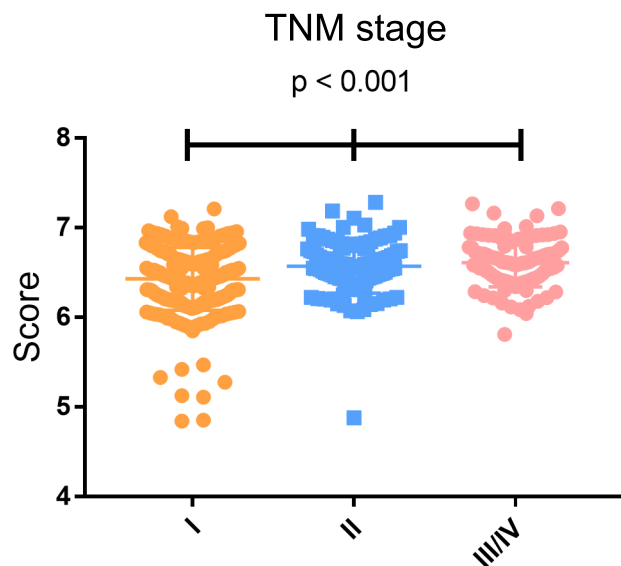

Supplement: Supplementary Figure 1 — Differences of risk score in subgroup of TCGA cohort. (A) age; (B) T stage; (C) N stage; (D) M stage; (E) sex; (F) TNM stage. [file DataSheet_1.pdf]

A

sex

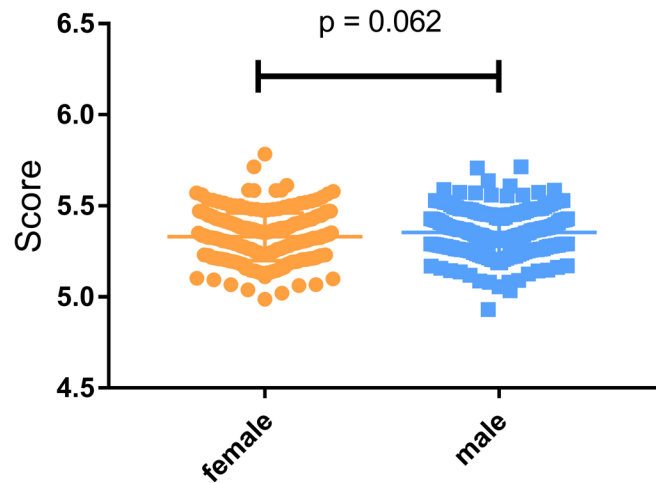

B

age

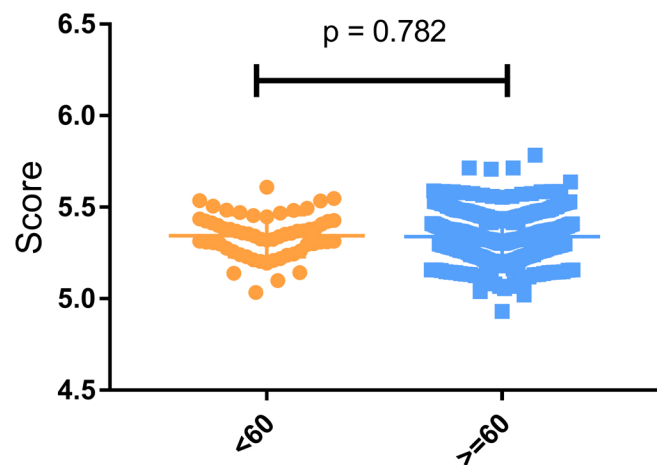

C

TNM stage

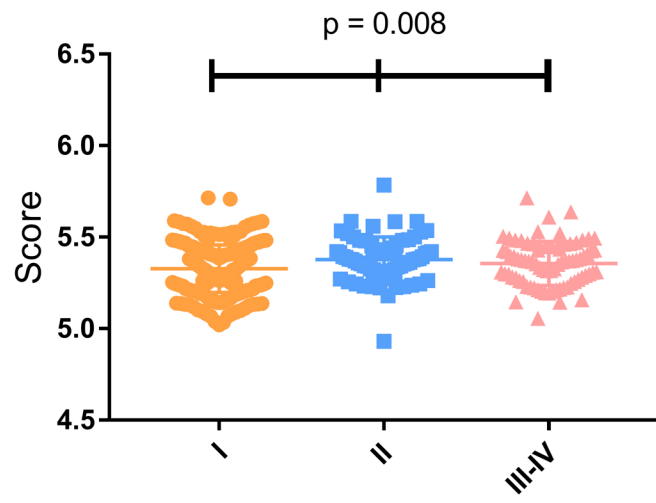

D

EGFR

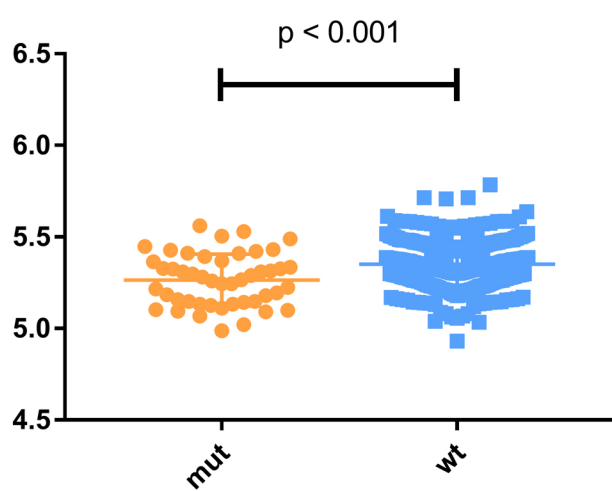

Supplement: Supplementary Figure 2 — Differences of risk score in subgroup of GEO cohort. (A) sex; (B) age; (C) TNM stage; (D) EGFR status. [file DataSheet_2.pdf]

A

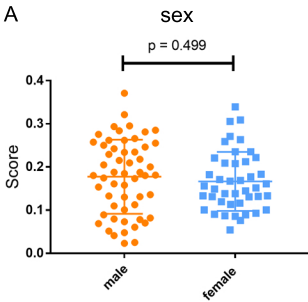

B

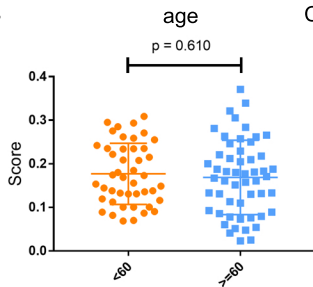

C

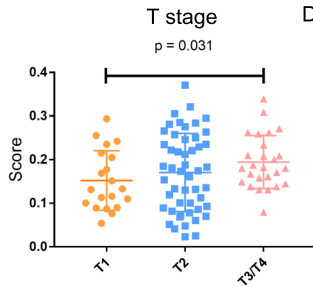

D

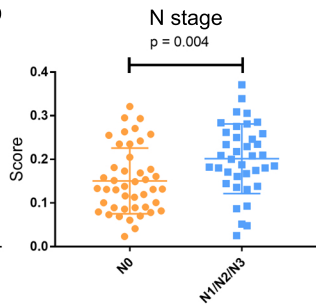

E

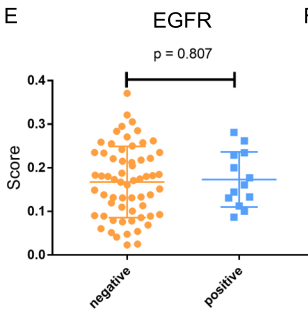

F

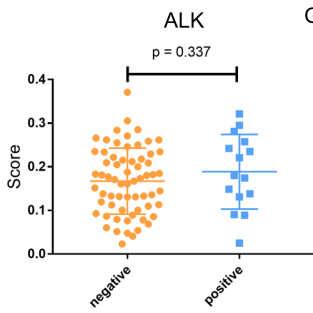

G

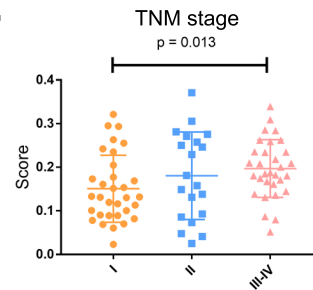

Supplement: Supplementary Figure 3 — Differences of risk score in subgroup of mIHC cohort. (A) sex; (B) age; (C) T stage; (D) N stage; (E) EGFR status; (F) ALK status; (G) TNM stage. [file DataSheet_3.pdf]
