# Supplementary material for: Identification of Key Genes Related to CD8+ T-Cell Infiltration as Prognostic Biomarkers for Lung Adenocarcinoma
Source: Front Oncol. 2021 Sep 28;11:693353. doi: 10.3389/fonc.2021.693353 (PMC8505972; doi:10.3389/fonc.2021.693353)
Supplement: Supplementary file 4 [file Table_1.docx]

Table 1. The clinical characteristics of TCGA-LUAD cohort

|  | Tumor | Normal |
| --- | --- | --- |
| Gender |  |  |
| Male | 219 | 21 |
| Female | 259 | 30 |
| age (years) |  |  |
| Mean | 65 | 66 |
| Median | 66 | 66 |
| TNM stage |  |  |
| Stage I | 266 | 28 |
| Stage II | 113 | 11 |
| Stage III | 74 | 10 |
| Stage IV | 25 | 2 |
| T stage |  |  |
| T1 | 163 | 17 |
| T2 | 252 | 32 |
| T3 | 45 | 2 |
| T4 | 15 | 0 |
| TX | 3 | 0 |
| M stage |  |  |
| M0 | 315 | 33 |
| M1 | 24 | 2 |
| MX | 139 | 16 |
| N stage |  |  |
| N0 | 318 | 28 |
| N1 | 84 | 10 |
| N2 | 64 | 10 |
| N3 | 2 | 0 |
| NX | 10 | 3 |
| EGFR status |  |  |
| Wild type | 184 | - |
| Mutate type | 74 | - |
| unknown | 220 | - |
