# Supplementary material for: Identification of Key Genes Related to CD8+ T-Cell Infiltration as Prognostic Biomarkers for Lung Adenocarcinoma
Source: Front Oncol. 2021 Sep 28;11:693353. doi: 10.3389/fonc.2021.693353 (PMC8505972; doi:10.3389/fonc.2021.693353)
Supplement: Supplementary file 5 [file Table_2.docx]

Table 2. The clinical characteristics of GEO cohort

|  | number |
| --- | --- |
| Gender |  |
| Male | 174 |
| Female | 219 |
| age (years) |  |
| Mean | 70 |
| Median | 70 |
| Smoking |  |
| Ever | 298 |
| Missing | 65 |
| Never | 30 |
| EGFR status |  |
| Mutate type | 47 |
| Wild type | 395 |
| TNM stage |  |
| I | 254 |
| II | 67 |
| III | 57 |
| IV | 15 |
