# Supplementary material for: Identification of Key Genes Related to CD8+ T-Cell Infiltration as Prognostic Biomarkers for Lung Adenocarcinoma
Source: Front Oncol. 2021 Sep 28;11:693353. doi: 10.3389/fonc.2021.693353 (PMC8505972; doi:10.3389/fonc.2021.693353)
Supplement: Supplementary file 6 [file Table_3.docx]

| Table 3. The clinical characteristics of mIHC cohort | | |
| --- | --- | --- |
| Characteristics | number | % |
| T stage |  |  |
| 1 | 20 | 20.4 |
| 2 | 50 | 51.0 |
| 3 | 21 | 21.4 |
| 4 | 5 | 5.1 |
| null | 2 | 2.0 |
| N stage |  |  |
| 0 | 44 | 44.9 |
| 1 | 18 | 18.4 |
| 2 | 14 | 14.3 |
| 3 | 6 | 6.1 |
| null | 16 | 16.3 |
| M stage |  |  |
| 0 | 97 | 99.0 |
| 1 | 1 | 1.0 |
| TNM stage |  |  |
| I | 32 | 32.7 |
| II | 20 | 20.4 |
| III | 30 | 30.6 |
| IV | 1 | 1.0 |
| null | 15 | 15.3 |
| Sex |  |  |
| Female | 43 | 43.9 |
| Male | 55 | 56.1 |
| Age |  |  |
| <60 | 45 | 45.9 |
| >=60 | 53 | 54.1 |
| Grade |  |  |
| I | 7 | 7.1 |
| II | 64 | 65.3 |
| III | 27 | 27.6 |
| EGFR |  |  |
| Negative | 62 | 63.3 |
| Positive | 13 | 13.3 |
| null | 23 | 23.5 |
| ALK |  |  |
| Negative | 65 | 66.3 |
| Positive | 15 | 15.3 |
| null | 18 | 18.4 |
